# Considering the healthcare needs of older people with multimorbidity: managing Alice

**DOI:** 10.15256/joc.2016.6.86

**Published:** 2016-05-11

**Authors:** Carolyn A. Chew-Graham, Jill Rasmussen, Neal Maskrey

**Affiliations:** ^1^Research Institute, Primary Care and Health Sciences, Keele University, Staffordshire, UK; ^2^Royal College of General Practitioners Clinical Lead Dementia, London; ^3^NHS England Clinical Lead Dementia South Region and Clinical Network South East, UK; ^4^School of Pharmacy, Keele University, Staffordshire, UK

**Keywords:** comorbidity, multimorbidity, multiple long-term conditions, patient-centred, primary healthcare

Managing elderly patients with multimorbidity can be challenging to clinicians, particularly those in primary care. We discuss the complexities and challenges in this editorial.

## Introducing Alice

Alice is 82 years old. She has type 2 diabetes mellitus and is on metformin – she only takes one 500 mg tablet twice a day as she “can’t stand” the abdominal discomfort and loose stools if she takes more, even though the general practitioner (GP) has told her she should take one with each meal. She takes levothyroxine, has asthma (which is managed with a Seretide^®^ inhaler) and hypertension (which is controlled with ramipril). For the pain in her knees and feet, which the GP says is due to “wear and tear”, she takes paracetamol regularly, but does not feel it works – and it might not, given the recent paper in the *Lancet* [1] – so she uses a rubefacient, which makes her feel better.

She was recently diagnosed with atrial fibrillation and, after much deliberation, agreed to start rivaroxaban. A year ago, Alice was given simvastatin by one of the practice nurses after having a blood pressure check, but often wonders why she needs to take this; and because the instructions are to take at night, Alice often forgets to take it.

Alice’s husband, Eric, died 2 years ago after a slow decline with dementia, having spent his final 6 months in a nursing home, which Alice can “never forgive herself for”. A short while after Eric’s death, Alice had gone to see the “lovely young doctor” at the practice who always had time for her, and seemed to understand how she felt about losing her husband. The doctor had started her on mirtazapine to help her to sleep.

Six months ago, Alice slipped on her doorstep when she was putting the milk bottle out (she does not drink much milk, but likes to see the milkman once a week to have a chat). Her neighbour called an ambulance and Alice was taken to the Emergency Department. She had sustained a Colle’s fracture and her left arm was put in plaster for 5 weeks. Thankfully, her neighbour helped with the shopping, and her daughter increased her visits from once to twice a week. Then one of the GPs rang her and told her she needed a special scan. Fortunately, the practice was able to organize hospital transport to take her for the scan, as she did not feel she could get the bus – her confidence had gone. Then a different GP phoned and told her that a prescription would be ready for her to start a tablet to strengthen her bones. The pharmacist spent ages telling her how to take it, and that she might have side effects. She has to admit that often on a Sunday, the day she should take it, she does not, as she fears the pain and discomfort that the tablet seems to cause.

## Considerations

It is helpful to think of Alice as a person who has multiple long-term conditions, or multimorbidity, where one problem is not more important than any of the others [2]. In today’s health service, multimorbidity is the norm, rather than the exception, and it is more helpful to think in terms of multimorbidity rather than “comorbidity”, in which there is usually an index condition (usually physical), and where the physical takes supremacy over the mental [2]. Multimorbidity also encourages a focus on the roles and responsibilities of the professional, rather than the patient’s needs and expectations. This is exacerbated by the context of a health (and social) care system organized around a single-disease approach, with single-disease guidelines (devised by the National Institute of Health and Care Excellence) and, in primary care, increasingly fragmented care where patients may be invited to attend for single-disease reviews [3]. Whilst there are benefits of proactive, planned care, particularly for people with long-term conditions, perhaps we should be moving back to a more “patient-centred” model [4] of care for patients with multimorbidity. The Ariadne principles can help us here [5] – spending time to enquire about what the patient’s priorities and goals are, rather than being driven by disease-focused processes and outcomes [6]. Muth *et al*. [5] list “practical hints” in three main areas:

- Assess potential interactions – the patient’s conditions and treatments, constitution, and context
- Elicit preferences and priorities – the patient’s most and least desired outcomes
- Individualize management to reach the negotiated treatment goals.

## Managing Alice

Ensuring that Alice sees a primary care clinician who she trusts is vital, yet we know that continuity of care in many GP practices has become more difficult to provide [7]. Alice might want to see the “lovely young doctor”, but this should be discussed fully, as Alice might not have wanted antidepressants, but took the prescription to please the doctor. The English contractual requirement to have a “named doctor” for patients over the age of 75 years may enable Alice to seek a doctor with whom she can develop and sustain a long-term, trusting relationship. Whilst most GP consultations are 10 minutes in the UK, many practices allow flexibility, and some investment “up front” may lead to reduced attendance in the future. So, a “double appointment” might allow the GP and Alice to talk through what Alice values in her life. Eliciting her health beliefs, for example, about how depression may impact her diabetes, or the risks and benefits of the statin or rivaroxaban, side effects of the alendronic acid and metformin, or how Alice feels about taking tablets in general, will open up the discussion. Eliciting what family and social support Alice has may support discussions about quality of life versus length of life.

An assessment of Alice’s mood and memory will help in decision-making about the need for an antidepressant, and the possibility of cognitive impairment. Alice’s history not only illustrates the challenges of multimorbidity but also the necessity and importance of being alert to her increased risk of dementia. Factors contributing to this risk include age and her physical illnesses diabetes and hypertension. Alice is also at increased risk of visual and/or hearing impairment, which the GP needs to consider in any review.

Perhaps Alice would prefer to be referred for some bereavement support, or sign-posted to a lunch club, yoga class, or knitting club. The need for primary care clinicians to identify third sector resources in their area is crucial to support a bio–psycho–social approach to patient care [8]. Consideration of “deprescribing” [9] may be helpful, particularly around the bisphosphonate and statin.

Alice’s view may be that she wants to do everything she can to treat her current conditions, whether they cause symptoms or not, and to prevent potential illness; but she may not. Being able to facilitate that full and frank discussion is, surely, a key role for the GP?

## Considerations for the practice

The other side of the coin of the challenges involved in managing Alice lead us to consider the opportunities for primary care to intervene in a positive way, which are summarized in Table 1. Offering continuity of care, even in larger practices, and when GPs are increasingly working part-time in the practice, to vulnerable groups of patients, such as those with multimorbidity, is something that practices should aspire to [7] – perhaps GPs (and nurse practitioners) might cover across the week in pairs, in order to enable a clinician who knows and to be available to patients with more complex conditions. Similarly, offering routine and regular appointments may reduce the risk of patients seeking unscheduled care. Sinnott *et al.* [11] report the acceptability of primary care clinicians of pre-arranged team meetings to discuss patients with multimorbidity and on multiple medications, and the usefulness of such reviews in reviewing prescribing decisions. Such clinical team meetings could extend to discuss principles around the management of patients with multimorbidity and the appropriateness of single-disease guidelines. Liaison with prescribing teams in Clinical Commissioning Groups and with Specialist care clinicians, and development of protocols and patient pathways across primary and specialist care, would ensure rationale prescribing across the primary/specialist interface.

**Table 1:** Opportunities for primary care in managing a patient with multimorbidity.

•	Named general practitioner
•	Continuity of care
•	Offering double appointments
•	Ensuring that the practice is accessible to, and can support, the needs of people with communication problems [10]
•	Collaboration and liaison between primary care professionals, across the primary/specialist interface, and including the patient
•	Integrating long-term condition reviews (ensuring that they are not disease-specific, but focused on the patient’s concerns and goals) [5]
•	Agreement amongst practice clinicians about managing a patient when single-disease guidelines may not be helpful
•	Regular meetings to discuss patients with multimorbidity and polypharmacy [11]

Finally, the importance of integration of care across settings and over time has been highlighted as key to effective management of people with complex needs [12] – all eyes are on Greater Manchester, where the health and social care budget has been merged, and with a key role for the local councils, to see the impact of such integration [13].
